# Oligofructose supplementation during pregnancy and lactation impairs offspring development and alters the intestinal properties of 21-d-old pups

**DOI:** 10.1186/1476-511X-13-26

**Published:** 2014-02-05

**Authors:** Laís Vales Mennitti, Lila Missae Oyama, Juliana Lopez de Oliveira, Ana Claudia Losinskas Hachul, Aline Boveto Santamarina, Aline Alves de Santana, Marcos Hiromu Okuda, Eliane Beraldi Ribeiro, Claudia Maria da Penha Oller do Nascimento, Luciana Pellegrini Pisani

**Affiliations:** 1 Programa de Pós Graduação Interdisciplinar em Ciências da Saúde, Universidade Federal de São Paulo, Santos, SP, Brazil; 2 Departamento de Biociências, Instituto de Saúde e Sociedade, Universidade Federal de São Paulo, Rua Silva Jardim, 136, Térreo, Vila Mathias, Santos, SP, Brazil; 3 Departamento Fisiologia, Disciplina de Fisiologia da Nutrição, Escola Paulista de Medicina, Universidade Federal de São Paulo, Rua Botucatu, 862, 2° andar, Vila Clementino, São Paulo, SP, Brazil

**Keywords:** Hydrogenated vegetable fat, Oligofructose, Corporal composition, Lipopolysaccharides, Lactobacillus spp, Pregnancy, Lactation

## Abstract

**Background:**

Previously, we showed that the intake of trans fatty acids during pregnancy and lactation triggers a pro-inflammatory status in the offspring. On the other hand, prebiotics can alter the intestinal environment, reducing serum lipopolysaccharides (LPS) concentrations. This study evaluated the effect of the oligofructose 10% diet supplementation in the presence or absence of hydrogenated vegetable fat during pregnancy and lactation on the development, endotoxemia and bacterial composition of 21-d-old offspring.

**Methods:**

On the first day of pregnancy rats were divided into four groups: control diet (C), control diet supplemented with 10% oligofructose (CF), diet enriched with hydrogenated vegetable fat, rich in TFA (T) or diet enriched with hydrogenated vegetable fat supplemented with 10% oligofructose (TF). Diets were maintained during pregnancy and lactation. At birth, 7th, 14th and 21th, pups were weighed and length was measured. Serum concentrations of LPS and free fatty acids (FFA) were performed by specific kits. Bacterial DNA present in faeces was determined by real-time PCR. Data were expressed as mean ± standard error of the mean and the statistical analysis was realized by ANOVA two-way and ANOVA for repeated measures. p < 0.05 was considered significant.

**Results:**

We observed that the oligofructose (10%) supplementation during pregnancy and lactation reduced body weight, body weight gain, length and serum FFA in the CF and TF group compared to C and T group respectively, of the 21-day-old offspring, accompanied by an increase in serum LPS and genomic DNA levels of lactobacillus spp. on faeces of the CF group in relation to C group.

**Conclusion:**

In conclusion, dam’s diet supplementation with 10% of oligofructose during pregnancy and lactation, independent of addition with hydrogenated vegetable fat, harms the offspring development, alters the bacterial composition and increases the serum concentrations of lipopolysaccharides in 21d-old pups.

## Background

Maternal nutrition during pregnancy and lactation plays a crucial role in the development foetal and newborn until adulthood, possibly influencing foetal “programming” by epigenetic modifications that can alter gene expression and permanently affect the structure and function of several organs and tissues, thereby inducing phenotypic changes [1-4]. Recently, the concept of foetal “programming” has been applied to examine possible beneficial or adverse influences of the maternal nutritional supply to the foetus and the newborn until later life [5].

During critical periods of foetal development, inadequate maternal nutrition may alter the physiologic and morphologic development of the foetus and the newborn, increasing the individual’s susceptibility to develop metabolic diseases in adulthood, such as cardiovascular disease, diabetes and hypertension [1-4]. A positive correlation between the development of metabolic disease and the production of pro-inflammatory cytokines is known [6,7].

Previous studies from our laboratory demonstrated that the maternal intake of hydrogenated vegetable fats that are rich in trans fatty acids (TFA) during pregnancy and lactation triggers changes in the lipid metabolism and decreases serum levels of adiponectin in 21-d-old pups. These findings were accompanied by increases in TNF-α gene expression and the protein expression of TRAF-6 (TNF receptor-associated factor 6) in the adipose tissue [8,9]. Furthermore, the authors reported that the consumption of TFA during lactation induces the development of metabolic disorders, including insulin resistance and the increased gene expression of plasminogen activator inhibitor type-1 (PAI-1) in the adipose tissue of the adult offspring [10,11]. PAI-1 is a pro-inflammatory adipokine produced mainly, in the visceral adipose tissue and vascular endothelium under influence of TNF-α, insulin, free fatty acids (FFAs) and glucocorticoids [12,13]. Elevated PAI-1 serum concentrations (eg. in obesity) are associated with pro-thrombotic effects, which increase the risk for cardiovascular disease [13,14].

Complementary results by Pimentel et al*.* showed that the increase in hypothalamic concentrations of IL-6, TNF-α and IL1-β contributed to the hypothalamus inflammation and impaired satiety control of 90-d-old pups from dams fed a diet rich in TFA during pregnancy and lactation [15].

On the other hand, several studies in humans and animals suggest that prebiotics and dietary fibres, such as oligofructose (OF), fructooligosaccharides (FOS) and inulin, can influence the glucose and lipid metabolism, specifically by reducing the serum concentrations of glucose, triacylglycerol (TG) and cholesterol [16-19]. OF, FOS and inulin are members of the inulin-type fructans group and are naturally present in many fruits and vegetables, including onions, bananas, artichokes, garlic and leeks [20-22]. Particularly, OF is a combination of “non-digestible” oligosaccharides commonly obtained by the partial enzymatic hydrolysis of chicory root inulin and is linked by β (2 → 1) linkages of fructosyl units, sometimes ending with a glucosyl unit [16,20,21]. OF and FOS are considered synonyms for inulin-type fructans with a maximum degree of polymerisation (DP) of less than 10 [16].

Furthermore, it is known that the human gastrointestinal tract enzymes only recognise α-glycosidic bonds; therefore OF and other inulin-type fructans that bear a β configuration are hydrolysed and fermented by colonic microbiota, with health benefits to the host [20,22-25]. Thus, inulin-type fructans are considered dietary fibres and prebiotics [20,23,26].

Mammal’s intestine has a microbial community consisting for approximately 10^12^ bacteria per g of intestinal content and its composition may vary according to some factors, such as: dietary nutrients, diseases and antibiotics therapy [23,27,28]. The mammal’s intestinal microbiota is composed for bacteria belonging to three phyla: gram-negative Bacteroidetes(eg Bacteroides), gram-positive Actinobacteria (eg Bifidobacteria) and gram-positive Firmicutes (eg. Lactobacillus, Clostridium, Bacillus and Mycoplasma) [27,29]. The phyla Firmicutes and Bacteroidetes are numerically dominant in the human intestine [27]. Kim et al. demonstrated an increase of Firmicutes, accompanied by a decrease of Bacteroidetes and Bifidobacteria in mice fed high-fat diet for 8 weeks [30].

Hyperlipidic diets, especially those rich in saturated fatty acid, increase endotoxemia through the translocation of lipopolysaccharides (LPS) found in the external cellular membranes of gram-negative intestinal bacteria [31]. LPS-induced TLR-4 (Toll-like receptor 4) activation contributes to systemic inflammation by inducing the secretion of pro-inflammatory cytokines [32]. Likewise, elevated plasma concentrations of FFAs are capable of utilising the TLR-4 pathway and activating the NF-kB pathway to induce pro-inflammatory cytokine expression [33-35]. In contrast, end products of prebiotic fermentation by the colonic bacteria, especially short chain fatty acids (SCFA - acetate, propionate and butyrate), can alter the intestinal environment through the decrease of colonic pH and the alteration of the bacterial population (mainly bifidobacteria and lactobacillus) and intestinal permeability, thereby reducing the migration of LPS to blood circulation [22,32,36].

Maternal intake of prebiotics and dietary fibres during pregnancy and lactation is considered important and beneficial for the mother and the offspring, from birth through later life. In particular, butyrate, one of the end products of oligosaccharide fermentation, is a histone deacetylase inhibitor, which can reactivate silent genes by epigenetic modifications. These effects on gene activity would be beneficial in the long term [37,38]. Additionally, the authors suggest that maternal intestinal microbiota function directly in the bacterial colonisation and intestinal properties of newborns and infants because human maternal milk presents a large variety and amount of oligosaccharides at concentrations ranging from 10 to 20 g/L [39,40]. It is also believed that some strains of bacteria present in the maternal gut are transferred from the mother to the newborn through the maternal milk [41]. The aim of the present study was to evaluate the effect of a dietary supplementation of 10% OF during pregnancy and lactation in the presence or absence of hydrogenated vegetable fat on the development, endotoxemia and bacterial composition of 21-d-old offspring.

## Materials and methods

### Animals and treatments

The experimental research committee of the Universidade Federal de São Paulo approved all procedures for the care of the animals used in this study and followed international recognised guidelines (CEUA protocol n°737014). The rats were kept under controlled conditions of light (12-h light/12-h dark cycle with lights on at 07:00) and temperature (24 ± 1°C), with *ad libitum* water and food. Three-month-old female Wistar rats (four animals in each group) were left overnight to mate, and copulation was verified the following morning by the presence of sperm in vaginal smears.

On the first day of pregnancy, the dams were isolated in individual cages and sequentially divided into four groups, each receiving one of four diets: a control diet (C diet, C group), a control diet supplemented with oligofructose (CF diet, CF group), a diet enriched with hydrogenated vegetable fat (T diet, T group) or a diet enriched with hydrogenated vegetable fat supplemented with oligofructose (TF diet, TF group). The diets were maintained throughout pregnancy and lactation. At birth, pups remained in the same group as the mother. The four diets were prepared according to the recommendations of the American Institute of Nutrition (AIN-93G) [42,43] and bore a similar calorific and lipid content. The source of lipids for the C and CF diets was soybean oil, and the principal source for the T and TF diets was partially hydrogenated vegetable fat, which is rich in TFAs. The CF and TF diets were prepared by adding 100 g/kg of oligofructose to the diet (Orafti P95, Pemuco, Chile). According to the manufacturer, the OF used in this study is a mixture of oligosaccharides extracted from chicory root. These oligosaccharides are composed of fructose units connected by ß (2–1) links, and a glucose unit terminates a few of these molecules. The DP of oligofructose in this supplement ranges between 2 and 8. The centesimal composition of the diets is presented in Table 1. Pisani et al. have previously described the fatty acid profile of C and T diets [8].

**Table 1:** Composition of the control diet, control diet supplemented with oligofructose, diet enriched with trans fatty acids and diet enriched with trans fatty acids supplemented with oligofructose according to AIN-93

			**Diet (g/100 g)**	
Ingredient	C	CF	T	TF
Casein*	20.0	20.0	20.0	20.0
L-cystine†	0.3	0.3	0.3	0.3
Cornstarch†	62.0	52.0	62.0	52.0
Soybean oil‡	8.0	8.0	1.0	1.0
Hydrogenated vegetablefat$	-	-	7.0	7.0
Butylhydroquinone†	0.0014	0.0014	0.0014	0.0014
Mineral mixture§	3.5	3.5	3.5	3.5
Vitamin mixture#	1.0	1.0	1.0	1.0
Cellulose†	5.0	5.0	5.0	5.0
Choline bitartrate†	0.25	0.25	0.25	0.25
Oligofructose£	-	10.0	-	10.0
Energy (kcal/g)	4.0	4.0	4.0	4.0

*Casein was obtained from Labsynth, São Paulo, Brazil.

†L-cystine, cornstarch, butylhydroquinone, cellulose and choline bitartrate were obtained from Viafarma, São Paulo, Brazil.

‡Oil was supplied from soybean (Lisa/Ind. Brazil).

$Hydrogenated vegetable fat was supplied from Unilever, São Paulo, Brazil.

§Mineral mix 9 mg/kg diet): calcium, 5000; phosphorus, 1561; potassium, 3600;sodium, 1019; chloride, 1571; sulfur, 300; magnesium, 507; iron, 35; copper, 6.0; manganese, 10.0; zinc, 30.0; chromium, 1.0; iodine 0.2; selenium, 0.15; fluoride, 1.00; boron, 0.50; molybdenum, 0.15; silicon, 5.0; nickel, 0.5; lithium, 0.1; vanadium, 0.1 (AIN-93G, mineral mix, Rhoster, Brazil).

#Vitamin mix (mg/kg diet): thiamin HCL, 6.0, riboflavin, 6.0; pyridoxine HCL 7.0;niacin, 30.0; calcium pantothenate, 16.0; folic acid, 2.0; biotin, 0.2; vitamin B12,25.0; vitamin A palmitate 4000 IU; vitamin E acetate, 75; vitamin D3, 1000 IU; vitamin KI, 0.75. (AIN-93G, vitamin mix, Rhoster, Brazil).

£Oligofructose (P95) was manufactured by Orafti (Pemuco, Chile) and was obtained by Viafarma, São Paulo, Brazil.

On the day of delivery, considered day 0 of lactation, litter sizes were adjusted to eight pups each. The pups were weighed and measured (naso-anal length) at birth and on postnatal days 7, 14 and 21.

### Experimental procedures

The pups were euthanized by decapitation on postnatal day 21. The animals were not fasted to avoid weaning stress. Trunk blood was collected and then immediately centrifuged at 2500 rpm for 15 minutes, and the serum was separated and stored at -80°C for the determination of lipopolysaccharides (LPS) and free fatty acids (FFA). The retroperitoneal white adipose tissue (RET) and liver were isolated, weighed, immediately frozen in liquid nitrogen and stored at -80°C. The gut and faecal content were removed and separated into portions, the cecum and colon, and were immediately placed in liquid nitrogen for the subsequent analysis of the colon bacterial DNA by real-time PCR (RT-PCR).

### Serum determination of lipopolysaccharides and free fatty acids

The concentration of LPS in serum was analysed using the Limulus Amebocyte Lysate (LAL) assay, a quantitative chromogenic test for detecting endotoxins (LAL QCL-1000 assay, Lonza, Walkersville, MD, USA). Serum samples were diluted 10 times with pyrogen-free water and incubated in pyrogen-free tubes at 75°C for 5 minutes. All the materials used in the test were initially autoclaved to render them pyrogen-free and to avoid interference in the test. The standard curve used in the assay was generated with known concentrations of LPS of the strain *Escherichia coli* O111:B4. The free fatty acids in the serum were determined with a 96-well Serum/Plasma Fatty Acid Kit Non-Esterified Fatty Acids 500 Point Detection Kit (Zenbio Inc., Research Triangle Park, NC, USA), following a 20-fold dilution of the samples. The manufacturer’s recommendations, which are listed in the protocols accompanying the product, were followed for the analysis.

### Genomic DNA extraction from faecal samples and real-time polymerase chain reaction

Genomic DNA from faecal samples of colon was extracted with the QiagenQIAmp DNA Stool Minikit (Qiagen, Valencia, CA, USA), according to the manufacturer’s recommendations. The DNA concentration per microlitre was measured using a spectrophotometer, NanoDrop ND-1000 (NanoDrop Technologies Inc., Wilmington, EUA), and the readings were acquired at wavelengths of 260, 280 and 230 nm. The purity was estimated by the 260/280 nm ratio, which must range between 1.8 and 2.0 for nucleic acids. All samples were maintained at -80°C.

Lactobacillus spp. was quantified by RT-PCR. Relative levels of lactobacillus spp. DNA were quantified in real time, using a SYBR Green primer in an ABI Prism 7500 Sequence Detector (both from Applied Biosystems, Foster City, CA, USA). Relative levels of the housekeeping gene of all bacteria were measured. The primers used were: lactobacillus spp., 5′- AGC AGT AGG GAA TCT TCC A-3′ (sense) and 5′-CAC CGC TAC ACA TGG AG-3′ (antisense), and all bacteria, 5′-TCC TAC GGG AGG CAG CAG T-3′ (sense) and 5′-GAC TAC CAG GGT ATC TAA TCC TGT T-3′ (antisense). The results were obtained using the Sequence Detector software (Applied Biosystems) and were expressed as the relative increase using the method of 2^-ΔΔCt^, described by Livak and Schmittgen [44].

### Statistical analysis

Data were submitted to quality tests, the Shapiro-Wilk (normality), Levenne (homogeneity) and/or Mauchly (sphericity) test, and were standardised to the Z score if necessary. Non-spherical data were corrected using F-values (Greenhouse-Geisser). The statistical significance of the differences between the means of the four group was assessed using a two-way analysis of variance (ANOVA) or ANOVA for repeated measure, followed by a Bonferroni post hoc test. All statistical tests were performed using the PASW Statistics 18 program, except for the comparison between C and TF groups, which was performed in the Stats Direct program using a one-way ANOVA and a Bonferroni post hoc test. The other functions were executed using the Microsoft Excel 2010 program. All results are presented as the means ± standard error of the mean (SEM), and differences were considered to be significant when *p* ≤ 0.05.

## Results

### Body weight, body weight gain and animal length

**Figure 1:**
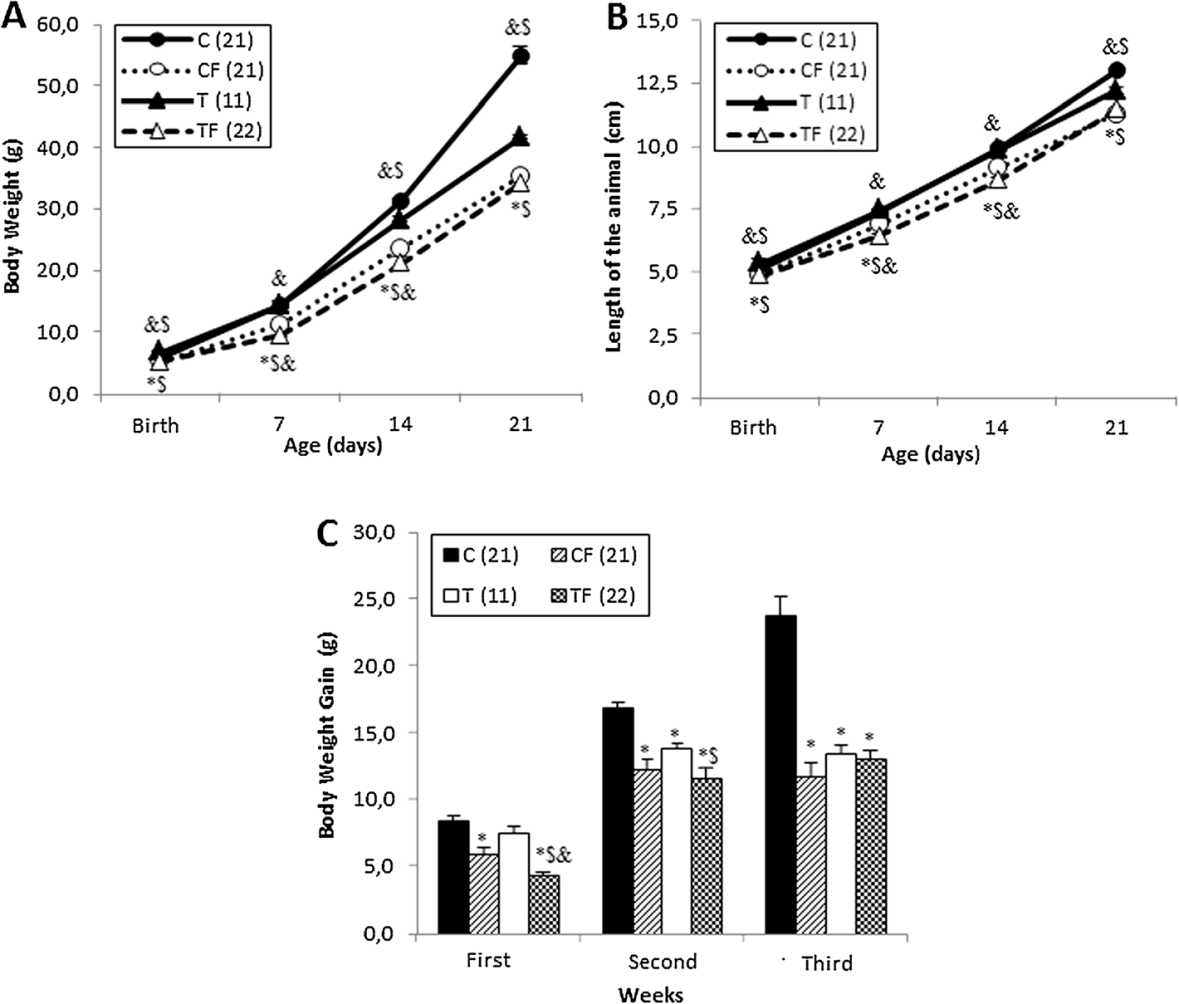
**Body weight (A), length of the animal (B) and body weight gain (C).** C –offspring of dams fed control diet; CF – offspring of dams fed control diet supplemented with oligofructose; T – offspring of dams fed diet enriched with hydrogenated vegetable fat; TF – offspring of dams fed diet enriched with hydrogenated vegetable fat supplemented with oligofructose. The number in parentheses refers to the sample value. Data are means ± SEMs. ^*^p ≤ 0.05 versus C. &p ≤ 0.05 versus CF. ^$^p ≤ 0.05 versus T. ^#^p ≤ 0.05 versus TF.

Over the entire period of treatment, the mean body weights (BWs) of the 21-d-old pups in the CF and TF groups were significantly lower than those of the C group (*p* ≤ 0.01 and *p* < 0.0001, respectively). Additionally, the TF group presented lower BWs than the T (*p <* 0.001) group during all of the experimental treatments. At birth, the BWs of the T group were significantly higher compared to the C (*p* = 0.001) group; however, by the second and third weeks of treatment, the C group had higher BWs compared to the T group (*p* ≤ 0.002). By postnatal days 7 and 14, the BW in the TF group was lower than the CF group (*p* ≤ 0.003) (Figure 1A).

Similarly, during all of the treatments, the BW gain of the offspring in the CF and TF groups was significantly lower than the C group (*p* < 0.001 and *p* < 0.0001, respectively). During the first and second week, the 21-d-old pups of the TF group presented a lower BW gain compared to the T (*p* < 0.02) group. By the second and third week, body weight gain in the C group was significantly higher compared to the T (*p ≤* 0.003) group. Furthermore, the TF group presented a lower BW gain than the CF group (*p <* 0.001) during the first week of treatment (Figure 1C).

Figure 1B shows that in the CF and TF groups, the length of the 21-d-old pups during the entire treatment was significantly lower than the C group (*p* ≤ 0.002 and *p* < 0.0001, respectively). Likewise, the TF group presented lower lengths than the T group (*p* < 0.001) over the entire experimental period. At birth, the T group presented greater lengths compared to the C group (*p* = 0.004); however, at postnatal day 21, the length of the C group was significantly higher in relation to the T group (*p* < 0.001). Finally, by postnatal days 7 and 14, the TF group presented smaller lengths than the CF group (*p* ≤ 0.003) (Figure 1B).

### Relative weight of tissues

The relative RET weight in the CF and T groups was significantly lower than the C group (*p* < 0.001). Moreover, the TF group presented a lower relative RET weight compared to the CF (*p* = 0.003) and C (*p* < 0.0001) groups (Table 2).

**Table 2:** Relative weight of retroperitoneal adipose tissue and liver in 21d-old pups

**Relative weight (g/100 g body weight)**	**C (n = 13)**	**CF (n = 15)**	**T (n = 9)**	**TF (n = 13)**
**RET**	0,376 ± 0,028	0,256 ± 0,028^*#^	0,152 ± 0,022^*^	0,156 ± 0,006^*^^&^
**LIVER**	3,562 ± 0,074	3,424 ± 0,128	3,792 ± 0,068	3,315 ± 0,088^*$^

C – offspring of dams fed control diet; CF – offspring of dams fed control diet supplemented with oligofructose; T – offspring of dams fed diet enriched with hydrogenated vegetable fat; TF – offspring of dams fed diet enriched with hydrogenated vegetable fat supplemented with oligofructose. The number in parentheses refers to the sample value. Data are means ± SEMs. ^*^p ≤ 0.05 versus C. ^&^p ≤ 0.05 versus CF. ^$^p ≤ 0.05 versus T. ^#^p ≤ 0.05 versus TF.

The relative weight of the liver in the TF group was significantly lower than the T (*p* = 0.003) and C (*p* = 0.041) groups (Table 2).

### Serum concentration of lipopolysaccharides and free fatty acids

**Figure 2:**
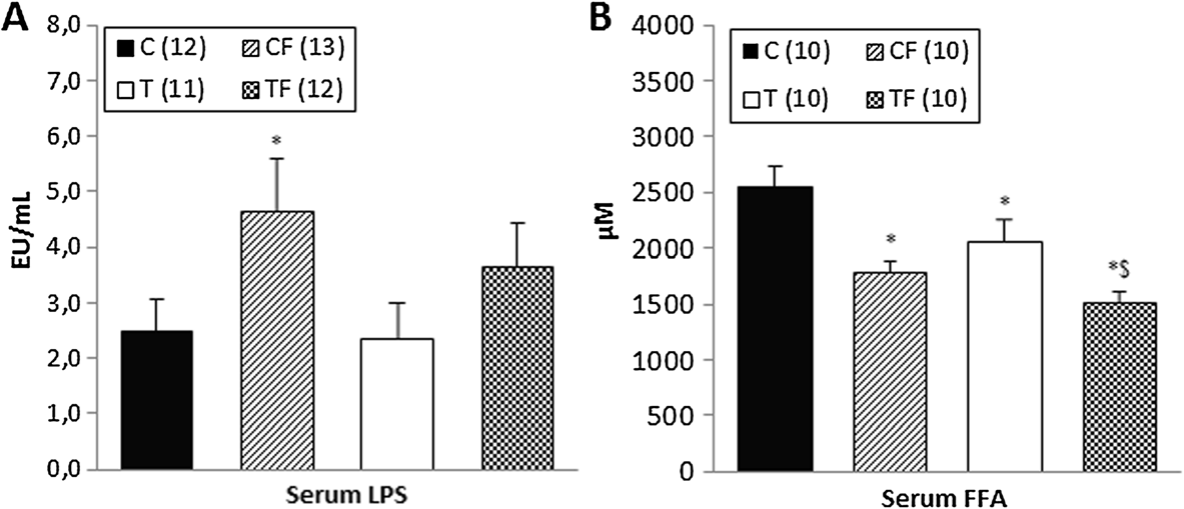
**Serum concentration of lipopolysaccharides (LPS) (A) and serum concentration of free fatty acids (FFA) (B).** C – offspring of dams fed control diet; CF –offspring of dams fed control diet supplemented with oligofructose; T – offspring of dams fed diet enriched with hydrogenated vegetable fat; TF – offspring of dams fed diet enriched with hydrogenated vegetable fat supplemented with oligofructose. The number in parentheses refers to the sample value. Data are means ± SEMs. ^*^p ≤ 0.05 versus C. &p ≤ 0.05 versus CF. ^$^p ≤ 0.05 versus T. ^#^p ≤ 0.05 versus TF.

The serum concentration of LPS in the CF group was significantly higher than the C group (*p* = 0.05) (Figure 2A).

Figure 2B shows that the FFA serum concentration in the CF, T and TF groups was lower compared to the C (*p* = 0.001, *p* = 0.03 and *p* < 0.0002, respectively) group. Further, the FFA serum concentration in the TF group was lower than the T group (*p* = 0.018).

### Levels of lactobacillus spp. in colon

**Figure 3:**
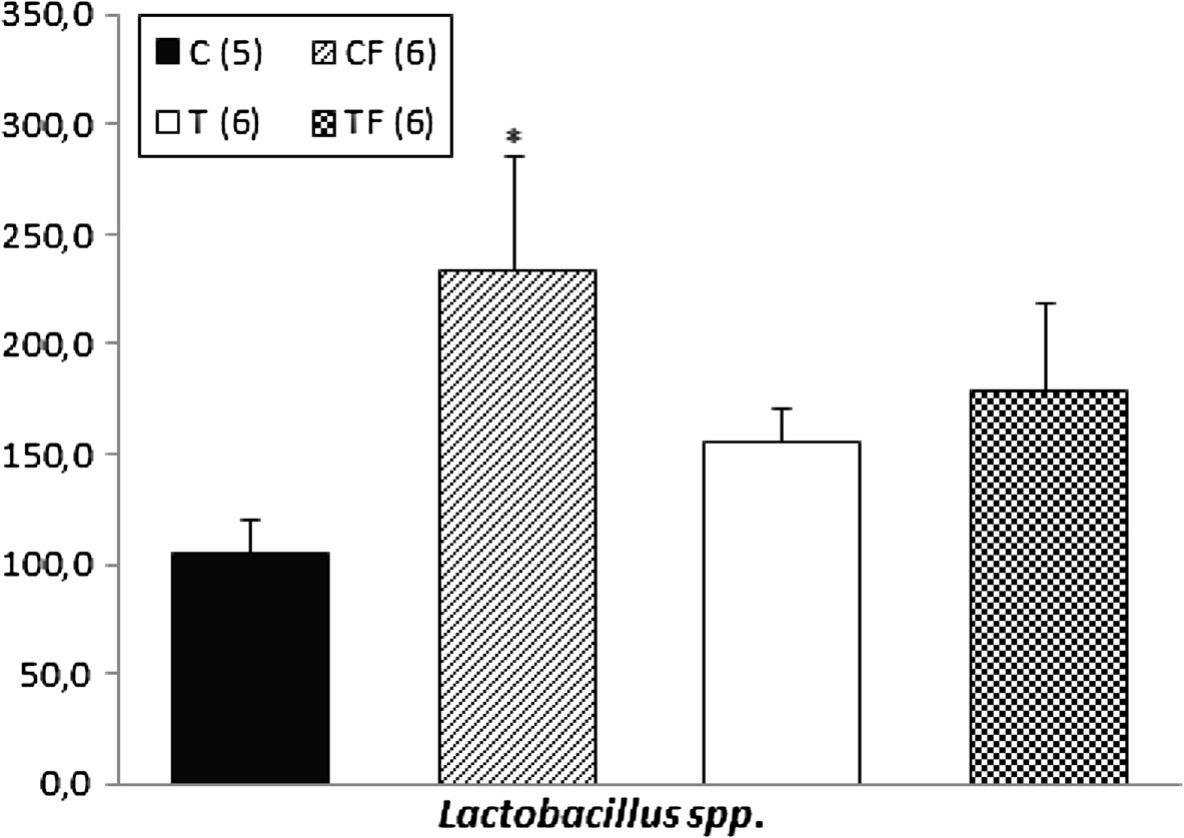
**Lactobacillus spp. genomic DNA levels on fecal content in colon.** C – offspring of dams fed control diet; CF – offspring of dams fed control diet supplemented with oligofructose; T – offspring of dams fed diet enriched with hydrogenated vegetable fat; TF – offspring of dams fed diet enriched with hydrogenated vegetable fat supplemented with oligofructose. The number in parentheses refers to the sample value. Data are means ± SEMs. Results are expressed in arbitrary units, stipulating 100 as the control value. ^*^p ≤ 0.05 versus C. &p ≤ 0.05 versus CF. ^$^p ≤ 0.05 versus T. ^#^p ≤ 0.05 versus TF.

The genomic DNA levels of the lactobacillus spp. in the faecal content of the colon of the CF group were 2.23-fold higher than the C group (*p* = 0.02) in 21-d-old offspring (Figure 3) and also in the TF group compared with the C group; however, this difference was not significant.

## Discussion

In the present study, supplementing the dam’s diet with 10% oligofructose during pregnancy and lactation decreased the body weight, body weight gain, length, relative weight of tissues and serum free fatty acids, which was accompanied by an increase in the serum concentration of lipopolysaccharides and lactobacillus spp. genomic DNA levels in the colon of the 21-d-old pups.

These results indicate that the supplementation of the dam’s diet with high amounts of oligofructose (10%) during pregnancy and lactation adversely affects the development and increases endotoxemia, possibly by bacterial translocation in the offspring.

The 21-d-old pups of the CF and TF groups presented lower body weights and lengths compared to the C and T groups (Figure 1), which were accompanied by a reduction in the free fatty acid serum concentration (Figure 2) and relative weights of the RET and liver (Table 2). Additionally, a lower body weight gain was also observed in the offspring of the CF group throughout the entire experimental period and in the TF group during the first and second weeks of treatment (Figure 1).

A literature review presents a limited and controversial picture of the effects of high-fibre diets during pregnancy and lactation. Corroborating our results, Carabin and Flamm reported a delay in the growth of pups from dams fed with 20% FOS diet during pregnancy and lactation [16]. On the other hand, previous studies did not demonstrate the negative effects of FOS supplementation during pregnancy on offspring development [16,45]. Furthermore, Pisani et al. showed that trans fatty acid intake during pregnancy and lactation did not modify the body weight of the pups during the entire period of lactation [10]. Thus, it could be concluded that high FOS supplementation during pregnancy and lactation could harm offspring development.

In our study, the birth weights of the offspring were affected by a trans fatty acid diet and 10% OF supplementation during pregnancy and lactation (Figure 1). In this regard, Hallam and Reimer reported that dietary supplementation with 21.6% prebiotic fibres (inulin and oligofructose mixture) during pregnancy and lactation decreased the birth weight of female offspring, whereas the birth weights of male offspring did not change. In the same study, the authors also demonstrated that there were no differences in the naso-anal length within male or female pups; however, the percentage of body fat at 4 weeks of age was lower in the high-fibre offspring [46]. Additionally, Maurer and Reimer did not observe any differences in the birth weight or body weight of the pups at postnatal days 7, 14 and 21 from dams fed a control, high-fibre (a combination of inulin and oligofructose) or high-protein diet during pregnancy and lactation [47]. Rodenburg et al. reported no difference in the body weight gain of 8-week-old rats fed a diet containing 6% FOS for 16 days [48]. Similarly, Parnell and Reimer showed that a high-fibre diet (10% and 20% of inulin and oligofructose) for 10 weeks did not influence the body weight and the fat mass in 8-week-old rats; however, the total liver weight decreased in the obese animals fed a diet supplemented with fibre prebiotics [49].

In accordance with results of corporal composition, we believe that the decrease in FFA serum concentration of the pups (Figure 2) reflects the reduction in the body weight (Figure 1) and RET-relative weight (Table 2) on the CF, T and TF groups compared to the C group. Adipose tissue is considered a major site of fatty acid storage in the body. In this regard, visceral fat depots, such as retroperitoneal adipose tissue, participate in the regulation of FFAs release to systemic circulation under several physiologic conditions [50]. Shadid and Jensen reported that weight loss by diet and exercise is associated with a decrease in FFA flux [51]. On the other hand, studies demonstrated that prebiotics are able to decrease the hepatic lipogenesis by a reduction in the activity and gene expression of hepatic lipogenic enzymes, such as fatty acid synthase (FAS) [18,52]. Kok et al*.* showed that oligofructose supplementation (100 g/kg of diet) for 30 days decreased the activity of the FAS enzyme in male Wistar rats [53]. In contrast, Parnell and Reimer demonstrated that high-fibre diets (10% and 20% of inulin and oligofructose, respectively) for 10 weeks increased FAS hepatic gene expression in JCR:La-cp rats [49]. Furthermore, studies demonstrated that dietary supplementation with prebiotic fibres during pregnancy and lactation (21,6%; inulin and oligofructose mixture) does not alter FAS gene expression in the liver and brown adipose tissue and does not reduce the FFA plasma concentration in the offspring [46,47].

Moreover, the changes in a pup’s body weight evolution and length of the CF and TF groups compared to the C and T groups (Figure 1) accompanied by a reduction in the liver relative weight of the TF group compared to the T group (Table 2) are consistent with the hypothesis that the 10% oligofructose supplementation during pregnancy and lactation contributes to offspring malnutrition, most likely as a consequence of impaired somatic and morphologic development. Taken together, these results suggest that the amount and type of the ingested prebiotic as well as the treatment period and physiological conditions could influence the development of the animal.

Finally, our results established that the 21-d-old offspring of the CF group had a higher lipopolysaccharide serum concentration (Figure 2), accompanied by a 2.23-fold increase in lactobacillus spp. genomic DNA levels in the faecal content of the colon (Figure 3) compared to the C group.

The inulin-type fructans are known to selectively stimulate the growth and the activity of the lactobacillus present in the colonic microbiota, thereby modulating the intestinal environment through changes in intestinal permeability, bacterial composition and SCFA production and contributing to the reduction in the LPS serum concentration, which benefits the host’s health [32,54,55]. In fact, Parnell and Reimer reported an increase in the lactobacillus spp. levels of 8-week-old obese rats fed a high-fibre diet (20% of inulin and oligofructose) for 10 weeks [56]. Mangell et al*.* demonstrated that *Lactobacillus plantarum 299v* can reduce *Escherichia coli*-induced intestinal permeability [57]. Additionally, the authors showed a reduction in the bacterial translocation to the liver and mesenteric lymph node of the rats pretreated with *Lactobacillus plantarum 229v* for one week before intra-peritoneal injection of LPS [58]. Rodes et al*.* demonstrated that the administration of *Lactobacillus rhamnosus* and *Lactobacillus reuteri* in an *in vitro* human colonic microbiota model decreased LPS concentrations in a time-dependent manner [59].

On the other hand, in accordance with our data, Ten Bruggencate et al*.* showed that a low calcium diet supplemented with FOS (60 g/kg of diet) for two weeks stimulated the growth of lactobacilli on cecal and colonic mucosa, accompanied by an increase in the intestinal permeability and translocation of *Salmonella enteritidis* in 8-wk-old Wistar rats [60]. Similarly, Ten Bruggencate et al*.* also demonstrated that the daily FOS consumption (20 g/day) associated with lower calcium intake during two weeks by healthy men increased the number of faecal lactobacillus, faecal mucin excretion and total faecal lactic acid excretion. The authors suggested that the mucin secretion induced by rapid production of organic acids (lactate and SCFA), in response to excessive prebiotic fermentation, is related to the irritation and impairment of the intestinal barrier function [61].

Another study demonstrated a dose-dependent increase in the colonisation and translocation of *Salmonella enteritidis*, accompanied by a higher lactic acid concentration in cecal contents in the FOS supplemented group (6% and 3%) in 8-wk-old Wistar rats; however, no changes were reported in the number of faecal lactobacilli [62]. Likewise, Rodenburg et al*.* observed an increase in the intestinal permeability and in the mitochondrial gene expression in 8-week-old rats submitted to a 16-day diet, low in calcium and supplemented with FOS (6%). In this study, the authors proposed that the rapid FOS fermentation by the colonic microbiota, resulting in acid lactic accumulation, excessive organic acids production and decreased luminal pH, leads to acidification of the cellular cytoplasm and indirectly induces ATP-depletion, which consequently increases the expression of colonic mitochondrial genes that may be involved in the maintenance of the intestinal barrier because disrupted energy metabolism leads to increases in intestinal permeability [48].

Accordingly, we observed that 10% FOS supplementation triggered diarrhoea in dams during the treatment (data not shown), which may be associated with the calcium loss. Additionally, it is possible that the oligosaccharides present in maternal milk [39,40] and the maternal intestinal bacteria transferred to the offspring through the breast milk [41] may lead to excessive production and luminal accumulation of organic acids in the pup’s gut, altering intestinal permeability and bacterial composition in the 21-d-old offspring [48,60-62]. Thus, changes in the composition of the microbiota and increases in intestinal permeability, along with damage to the intestinal barrier integrity, can cause an increase in bacterial translocation and LPS serum concentration, resulting in TLR4-mediated inflammatory responses in the offspring [32].

In fact, we previously showed that 10% oligofructose supplementation during pregnancy and lactation increased the TNF-α content in the liver of pups in the CF group and IL-6 and TNF-α contents in RET of pups in the TF group, accompanied by a reduction in the serum adiponectin concentrations of the offspring in the CF, T and TF groups [63].

## Conclusion

In conclusion, supplementation with 10% oligofructose during pregnancy and lactation, in the presence or absence of hydrogenated vegetable fat harms offspring development and increases endotoxemia, most likely due to damage to the intestinal permeability, changes in colonic bacterial population and impairment of the intestinal mucosal barrier integrity, which promotes an increase in serum concentrations of lipopolysaccharides in 21-d-old pups. Further studies are needed to investigate the dose-dependent effects of oligofructose ingestion during gestation and lactation as well as on the development, metabolism and endotoxemia of the pups.

## Competing interests

The authors declare that they have no competing interests.

## Authors’ contribution

LVM - designed the study, carried out the experiments, performed the statistical analysis and drafted the manuscript. LMO- helped to carried out the experiments, revised and helped to draft the manuscript. JLO - participated in the design of the study helped to carried out the experiments. ACLH - participated in the design of the study helped to carried out the experiments. ABS - helped to carried out the experiments. AAS - helped to carried out the experiments. MHO – helped to perform the statistical analysis. EBR - helped to draft the manuscript. CMON - conceive the study, participated in its design, and helped to draft the manuscript. LPP - conceive the study, participated in its design, and helped to draft the manuscript. All authors read and approved the final manuscript.
